# Arbuscular Mycorrhizal Fungi Improve the Performance of Sweet Sorghum Grown in a Mo-Contaminated Soil

**DOI:** 10.3390/jof6020044

**Published:** 2020-03-31

**Authors:** Zhaoyong Shi, Jiacheng Zhang, Shichuan Lu, Yang Li, Fayuan Wang

**Affiliations:** 1 College of Agriculture, Henan University of Science and Technology, Luoyang 471023, China; shizy1116@126.com (Z.S.); zjc19930726@163.com (J.Z.); lu337918969@126.com (S.L.); ly732196@163.com (Y.L.); 2 Henan Engineering Research Center for Rural Human Settlement, Luoyang 471023, China; 3 Luoyang Key Laboratory of Symbiotic Microorganism and Green Development, Luoyang 471023, China; 4 College of Environment and Safety Engineering, Qingdao University of Science and Technology, Qingdao 266042, China; 5 Key Laboratory of Soil Resources and Environment in Qianbei of Guizhou Province, Zunyi Normal University, Zunyi 563002, China

**Keywords:** molybdenum, arbuscular mycorrhizae, heavy metal pollution, ecological restoration, phytoremediation, bioaccumulation

## Abstract

Arbuscular mycorrhizal fungi are among the most ubiquitous soil plant-symbiotic fungi in terrestrial environments and can alleviate the toxic effects of various contaminants on plants. As an essential micronutrient for higher plants, molybdenum (Mo) can cause toxic effects at excess levels. However, arbuscular mycorrhizal fungal impacts on plant performance and Mo accumulation under Mo-contamination still require to be explored. We first studied the effects of *Claroideoglomus etunicatum* BEG168 on plant biomass production and Mo accumulation in a biofuel crop, sweet sorghum, grown in an agricultural soil spiked with different concentrations of MoS_2_. The results showed that the addition of Mo produced no adverse effects on plant biomass, N and P uptake, and root colonization rate, indicating Mo has no phytotoxicity and fungitoxicity at the test concentrations. The addition of Mo did not increase and even decreased S concentrations in plant tissues. Arbuscular mycorrhizal inoculation significantly enhanced plant biomass production and Mo concentrations in both shoots and roots, resulting in increased Mo uptake by mycorrhizal plants. Overall, arbuscular mycorrhizal inoculation promoted the absorption of P, N and S by sweet sorghum plants, improved photosystem (PS) II photochemical efficiency and comprehensive photosynthesis performance. In conclusion, MoS_2_ increased Mo accumulation in plant tissues but produced no toxicity, while arbuscular mycorrhizal inoculation could improve plant performance via enhancing nutrient uptake and photochemical efficiency. Sweet sorghum, together with arbuscular mycorrhizal fungi, shows a promising potential for phytoremediation of Mo-contaminated farmland and revegetation of Mo-mine disturbed areas, as well as biomass production on such sites.

## 1. Introduction

Molybdenum (Mo) is a transition metal with low abundance in the lithosphere, but has wide applications in many economic sectors, such as alloy, electronic parts, lubricants, catalysts, and agricultural production [1]. It is also an essential nutrient for most organisms, and a component of some plant enzymes involved in oxidation and reduction reactions [2]. Among the micronutrients, Mo represents one of the scarcest trace elements in plant tissues [2]. Most plants contain Mo concentrations in the range of 0.8 to 5 mg/kg [3], but the critical values between deficiency and toxicity can vary from 0.1 to 1000 mg Mo per kg dry mass [4]. Mo concentrations can reach up to 1585 mg/kg and 1800 mg/kg in the leaf tissues of cotton and turnips respectively [5]. Although Mo is low in phytotoxicity, exposure to excess Mo (40 mg/L) can cause adverse impacts in plants, such as chlorosis and yellowing [6]. Particularly, excessive Mo in crops may pose potential health risks for humans and animals via food chains. For instance, herbages containing Mo concentrations of 10 to 20 μg/g can cause fatal molybdenosis in ruminants via inducing Cu deficiency (i.e., hypocuprosis) [7]. Hence, Mo content in the crops growing on Mo-contaminated sites needs to be addressed. 

Mo uptake by plants depends mainly on Mo forms and concentrations in soils, which can be increased by human activities such as mining [8] and sludge applications [9]. In Mo-mining affected soils, Mo concentrations can reach up to 1071.52 mg/kg [10] and 2903.91 mg/kg [11], respectively. Mo concentrations in the rice grains harvested from a Mo-mining impacted field ranged from 0.58 to 12.04 mg/kg [12]. The application of sludge with higher Mo concentrations increased the Mo uptake in forage grasses and legumes [9]. Consequently, Mo-contaminated crops may impose potential health risk for consumers. It is of great interest to study Mo bioaccumulation and phytotoxicity in plants, and phytoremediation of Mo-contaminated soil. 

Arbuscular mycorrhizal (AM) fungi (AMF) are among the most ubiquitous soil plant-symbiotic fungi in terrestrial environments, including sites contaminated by metallic and organic contaminants [13,14]. AMF have been shown to improve plant mineral nutrition and attenuate the toxicity of various contaminants to plants, thereby facilitating them to grow better on contaminated sites [13,14,15]. AM extraradical mycelia can facilitate the solution of non-available nutrients (most importantly P) in the soil, and deliver them to their host plants. Interestingly, the nutrients in the soil, especially P and S, can affect Mo uptake in plants. Plant uptake of Mo is generally elevated by soluble P but decreased by available S [16]. Given the above context, we hypothesize that, in Mo-contaminated soil, AMF may change plant growth and Mo uptake and accumulation directly or indirectly through regulating other nutrient uptake; these hypotheses still remain to be verified. 

Plants are known to have different tolerances to environmental stress. Sweet sorghum, the plant we used in our experiment, has wide adaptability and high resistance to stressful environments and can grow on marginal lands with various disadvantages, including heavy metal contamination [17] and salty soils [18]. Due to its high nutrition values and sugar-rich stalk, sweet sorghum is considered both an excellent forage crop and biofuel crop [19,20]. It is of great significance for bioenergy production to study the growth of sweet sorghum on contaminated sites. Hence, sweet sorghum may be potentially used for both the biomass production and ecological restoration of fragile environments such as Mo-impacted sites. 

To date, only one study reported AMF effects under Mo-contamination, and the preliminary results showed AMF increased Mo uptake by maize plants in a soil spiked with phytoavailable (NH_4_)_2_MoO_4_ [21]. However, soluble molybdate rarely exists in soil. Sulfide molybdenite (MoS_2_) is the principal component of commercially mined ore in China, and the most common contaminant generated by Mo processing and tailings. Hence, it is of significance to study toxicity, uptake, and bioaccumulation of Mo in plants exposed to MoS_2_ as influenced by AMF. Sweet sorghum is used as both an excellent forage and biofuel crop [22], and a phytoremediation plant [23]. If AMF increase the biomass production and Mo accumulation, mycorrhizal sweet sorghum can be used for both bioenergy production and phytoremediation of polluted sites. If AMF increase sweet sorghum growth but decrease Mo accumulation to a safe level, then they can be used for forage production.

Considering the importance of AMF in plant nutrition and tolerance, we hypothesize that AMF can improve plant performance of sweet sorghum in soil contaminated with MoS_2_. The objectives of our present experiment are to explore (1) the effects of excessive MoS_2_ on performance of sweet sorghum; and (2) AMF impacts on plant growth and Mo accumulation and the underlying mechanisms. Using an agricultural soil spiked with MoS_2_, we conducted a microcosm experiment to explore the growth and Mo uptake of sweet sorghum inoculated with or without AMF. Our ultimate aims are to verify whether AMF can improve the performance of sweet sorghum, and to know their potential for biomass production and revegetation or phytoremediation of MoS_2_-contaminted sites. 

## 2. Materials and Methods

### 2.1. Soil, Plant and AMF Inoculum

Loamy soil taken from a farmland was used for the pot culture experiment (Table 1). Prior to use, soil was sterilized for 60 min at 121 °C to eliminate indigenous AMF. Sweet sorghum (*Sorghum bicolor* (L.) Moench var. Yajin2) was selected as the target plant. Due to its high tolerance to infertility and drought, this cultivar is widely grown in China. Prior to sowing, seeds were surface-sterilized with NaClO solution. AM fungal strain *Claroideoglomus etunicatum* (formerly known as *Glomus etunicatum*) BEG168 was propagated for 12 weeks using maize plants in sterilized sand [24]. The inoculum comprised a mixture of sand, spores, mycelium, root fragments, and contained approximately 1000 spores per 100 g.

### 2.2. Experimental Set-up and Procedure

The contamination status of Mo was simulated by artificially mixing MoS_2_ into the soil. Based on our investigation that found Mo-contamination status can reach up to 5000 mg/kg in farmlands in the vicinity of local Mo mines, we set up a series of Mo levels, i.e., 0, 1000, 2000, 3000, 4000 and 5000 mg/kg, in our present experiment. An appropriate amount of MoS_2_ (analytical reagent grade) was thoroughly mixed into the soil to achieve the target Mo concentrations. To determine the effects of AMF, 100 g air-dried inoculum of *C. etunicatium* BEG168 was mixed into each pot containing 900 g soil to culture mycorrhizal plants, while an equal amount of sterilized AMF inoculum was used for non-inoculated treatments. Therefore, this was a 6 × 2 designed two-factor experiment. Four replicates were designed for each treatment.

Ten uniform seeds were grown in each pot. Seven seedlings were retained per pot 7d after seed emergence. All pots were randomly arranged in a plant cultivation chamber with a day/night (12/12 h) temperature of 28–30/23–26 °C (light intensity of 5000 lux) and a relative air humidity of 50–80%. Distilled water was irrigated to meet the plant’s requirement. 

### 2.3. Sample Analysis

Photo-induced transients of prompt fluorescence in leaves were measured using a M-PEA fluorometer (Multi-Function Plant Efficiency Analyser, Hansatech, UK) based on the procedure described by Strasser et al. [25] and Kalaji et al. [26]. The third leaf from the top of the plant was selected for measurement after 20 min of dark adaptation. Three leaves per pot were measured. The JIP-test parameters, calculated based on the previous definitions, were shown in Table 2 [25,27].

Plants were harvested and sampled after 4 months of growth. Fresh roots were subsampled for evaluation of root colonization rate. The remaining plant tissues were dried in an oven at 70 °C for 48 h for estimation of dry weights (DWs) and elemental analysis. 

Root colonization rate was estimated based on method proposed by Trouvelot et al. [28] after ink staining [29]. Briefly, fresh root samples were cleared in boiling 10% KOH solution for 3 min, acidized in vinegar for 5 min, and then stained for 3 min in a boiling ink (5%)-vinegar solution. Thirty stained root segments with lengths of about 1 cm were observed under higher magnification using a microscope to determine the frequency of mycorrhizal colonization in the root system. The dried plant materials were ground using a mortar and pestle and digested in a mixture of H_2_SO_4_ and H_2_O_2_. The concentrations of Mo and P in the digested solution were determined by ICP-OES (Optima 7300 DV, Perkin Elmer, Waltham, MA, USA). Subsamples of plant materials were taken for analysis of N and S concentrations, which were determined by dry combustion in an Elementar vario-macro C/N analyzer (Elementar Analysensysteme, GmbH, Hanau, Germany). 

### 2.4. Statistical Analysis

SPSS 22.0 software was used to analyze the data. The results were presented as means ± standard error (SE). A Duncan test was performed to compare statistically significant differences (*p* < 0.05) among means in different treatments. Two-way ANOVA analysis was conducted to test for the significance (*p* < 0.05 and *p* < 0.01) of the interaction between soil Mo concentrations and AM inoculation. Pearson correlation coefficients were calculated to analyze the relationship between Mo concentrations in soil and in plant tissues. 

## 3. Results and Discussion

### 3.1. Root Colonization 

**Figure 1:**
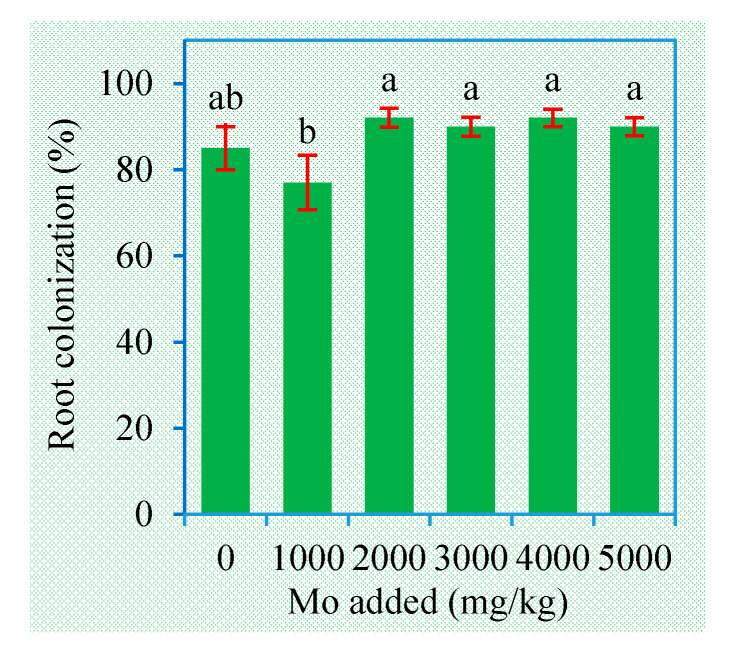
Root colonization rate (mean ± SE, *n* = 4) of the inoculated sweet sorghum exposed to different concentrations of Mo. Different letters over the bar represent significant differences between the means among different treatments using one-way ANOVA followed by Duncan’s multiple range test (*p* < 0.05).

Mycorrhizal colonization was not found in roots of the non-inoculated plants and thus not shown in Figure 1. Comparatively, the inoculated plants all had root colonization rates higher than 77%. Compared to the zero Mo concentration, additions of Mo had no significant effects on root colonization rates, and even some increasing effects at the concentrations higher than 1000 mg/kg. 

Due to their excellent tolerance to soil contaminants, AMF can survive in various sites impacted by mining activities, including coal, metallic and other mining sites [13]. In an abandoned Mo mine with 20 years of mining Mo and other heavy metals, abundant AMF were observed in both the soil and roots, and *C. etunicatum* was the most dominant species [30]. In a previous study, *C. etunicatum* BEG168 was shown to colonize maize plants exposed to an oxoanion molybdate (NH_4_)_2_MoO_4_ with concentrations up to 4000 mg Mo per kg soil [21], indicating a low toxicity of MoO_4_^2−^ to AMF. Our present experiment further confirmed that MoS_2_ did not influence the infectivity of this AM strain to sweet sorghum. MoS_2_ is a stable mineral with low solubility in soil, and the component Mo is low in bioavailability, which may account for its low or non-fungitoxicity. More importantly, Mo is an essential element for plant endosymbionts such as rhizobia and mycorrhizal fungi [31]. The addition of Mo can benefit the survival of rhizobial cells [32]. Likewise, Mo is expected to produce beneficial effects on AMF growth and colonization. 

### 3.2. Plant Biomass

**Figure 2:**
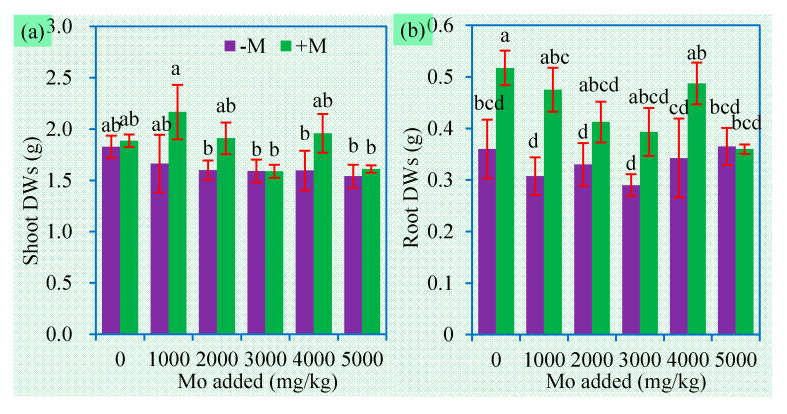
Shoot (**a**) and root (**b**) DWs (mean ± SE, *n* = 4) of sweet sorghum exposed to different concentrations of Mo. −M and +M represent non-AM inoculation and inoculation with *Claroideoglomus etunicatum* BEG168, respectively. Different letters over the bar represent significant differences between the means among different treatments using one-way ANOVA followed by Duncan’s multiple range test (*p* < 0.05). Two-way ANOVA results for shoot DWs: AM inoculation: *F* = 4.6 *, Mo: *F* = 1.1 ns, AM × Mo: *F* = 0.5 ns; two-way ANOVA results for root DWs: AM inoculation: *F* = 17.0 **, Mo: *F* = 1.4 ns, AM × Mo: *F* = 1.0 ns. Significance levels: * *p* < 0.05; ** *p* < 0.01; ns non-significant effect.

As an essential micronutrient for plants, Mo can produce “low-dose stimulation and high-dose inhibition” effects. Excess Mo can cause a series of side-effects on plants, but the phytotoxicity concentrations of Mo vary widely with plants and soil conditions [16,33]. Monocotyledonous plants like Gramineae usually have stronger resistance to Mo toxicity than dicotyledonous species [34]. Notwithstanding, excess soluble Mo can produce phytotoxic effects [35]. In our present experiment, both shoot and root DWs were not significantly affected by the addition of MoS_2_ (Figure 2). These findings indicate that Mo toxicity highly depends on its speciation and bioavailability. Our present study first showed that, unlike phytoavailable MoO_4_^2−^, insoluble molybdates such as MoS_2_ have low bioavailability and no phytotoxicity. 

Based on two-way ANOVA results, AM inoculation showed positive effects on plant growth, particularly on root DWs. On average, root and shoot DWs of the inoculated plants increased by 33% and 13% respectively, compared to the non-inoculated plants. AMF benefits on plant growth under Mo stress have been confirmed by Shi et al. [21]. We further evidenced AMF promoted plant growth in soil with high levels of MoS_2_. This implies potential applications of AMF in plant establishment and ecological restoration of the sites disturbed by Mo-mining. Meanwhile, AMF may facilitate biomass production of sweet sorghum on Mo mine areas. 

### 3.3. Mo Concentrations and Uptake in Plant Tissues 

**Figure 3:**
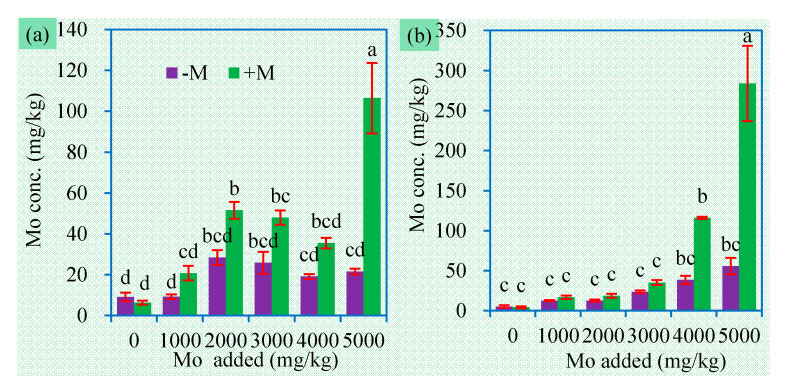
Shoot (**a**) and root (**b**) Mo concentrations (mean ± SE, *n* = 4) of sweet sorghum exposed to different concentrations of Mo. −M and +M represent non-AM inoculation and inoculation with *Claroideoglomus etunicatum* BEG168, respectively. Different letters over the bar represent significant differences between the means among different treatments using one-way ANOVA followed by Duncan’s multiple range test (*p* < 0.05). Two-way ANOVA results for shoot Mo concentrations: AM inoculation: *F* = 60.9 **, Mo: *F* = 23.3 **, AM × Mo: *F* = 13.0 **; two-way ANOVA results for root Mo concentrations: AM inoculation: *F* = 13.0 **, Mo: *F* = 13.4 **, AM × Mo: *F* = 6.4 **. Significance levels: * *p* < 0.05; ** *p* < 0.01; ns non-significant effect.

**Figure 4:**
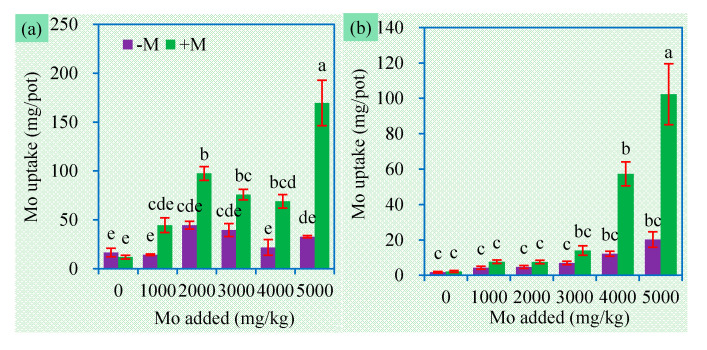
Shoot (**a**) and root (**b**) Mo uptake (mean ± SE, *n* = 4) of sweet sorghum exposed to different concentrations of Mo. −M and +M represent non-AM inoculation and inoculation with *Claroideoglomus etunicatum* BEG168, respectively. Different letters over the bar represent significant differences between the means among different treatments using one-way ANOVA followed by Duncan’s multiple range test (*p* < 0.05). Two-way ANOVA results for shoot Mo uptake: AM inoculation: *F* = 103.0 **, Mo: *F* = 26.5 **, AM × Mo: *F* = 15.4 **; two-way ANOVA results for root Mo uptake: AM inoculation: *F* = 43.3 **, Mo: *F* = 31.0 **, AM × Mo: *F* = 15.7 **. Significance levels: * *p* < 0.05; ** *p* < 0.01; ns non-significant effect.

Shoot and root Mo concentrations in plants receiving no Mo addition ranged from 4 to 9 mg/kg (Figure 3), which are slightly higher than the normal values (0.80 to 5.0 mg/kg) in common crops [3]. Comparatively, plants receiving additional Mo had much higher Mo concentrations and uptake in both shoots and roots (Figure 3, Figure 4). Root Mo concentrations and uptake always showed an increasing trend with the increase in Mo levels (Table S1). Although MoS_2_ is very stable in soil, it dissolves more readily at higher soil pH and redox potential [16]. We used a slightly alkaline soil with pH 7.32 and loamy texture. Thus, MoS_2_ may serve as a Mo sink to continuously release MoO_4_^2−^ for plants. 

Nonetheless, Mo concentrations in sweet sorghum did not exceed 300 mg/kg, which are much lower than the critical toxicity value of 500 mg/kg in most crop species [36]. This can partially explain why sweet sorghum did not exhibit toxic symptoms. Maize plants grown in soil spiked with (NH_4_)_2_MoO_4_ accumulated up to 800 and 3000 mg/kg Mo in their shoots and roots respectively, and displayed typical toxic symptoms [21]. Because both sweet sorghum and maize belong to Gramineous species with similar Mo requirements, the differences in Mo accumulation between them could be ascribed to the bioavailability of the Mo added. Thus, sweet sorghum could be grown in molybdenite-disturbed sites to achieve biomass production. 

More importantly, AM inoculation always enhanced Mo accumulation in both shoot and roots in soil added with MoS_2_ (Figure 3, Figure 4). This is similar to the findings that AMF increased Mo accumulation in maize plants from soil spiked with (NH_4_)_2_MoO_4_ [21]. AMF can facilitate the plant nutrient uptake of macronutrients and micronutrients. AMF extraradical hyphae, with a much greater surface area than plant roots, can absorb nutrients from where roots cannot reach and supply them for plants, leading to enhanced Mo acquisition by plants [37]. It is already recognized that plant Mo uptake is elevated by the presence of soluble P [38], while AMF have excellent ability to enhance the solution of insoluble P. The bioavailability of Mo usually positively correlates with soil pH [39], while AMF can increase higher soil pH to mediate the availability of toxic metals [40,41]. Of course, how AMF regulate Mo uptake and transport in the symbionts still remain to be elucidated. 

Generally, plant Mo concentrations are correlated positively with Mo levels in the plant growth substrate [42,43]. In our experiment, root Mo concentrations and shoot Mo concentrations in mycorrhizal plants showed positive correlations with soil Mo concentrations (Table 2). In soil added with 5000 mg/kg Mo, mycorrhizal plants accumulated 4 times more Mo than nonmycorrhizal plants (Figure 3). These findings lead to the following aspects regarding phytoremediation and crop production. Mycorrhizal plants may have a different survival strategy to deal with excessive Mo, and hence they are better candidate for phytoremediation of Mo-contaminated sites. High levels of Mo-contaminants such as MoS_2_ can also cause Mo accumulation and consequently toxicity in plants growing on Mo-contaminated sites. In Mo-contaminated farmland, Mo contents in crops, particularly in edible parts, should be monitored for safe crop production. 

### 3.4. Concentrations of P, N, and S in Plants

**Figure 5:**
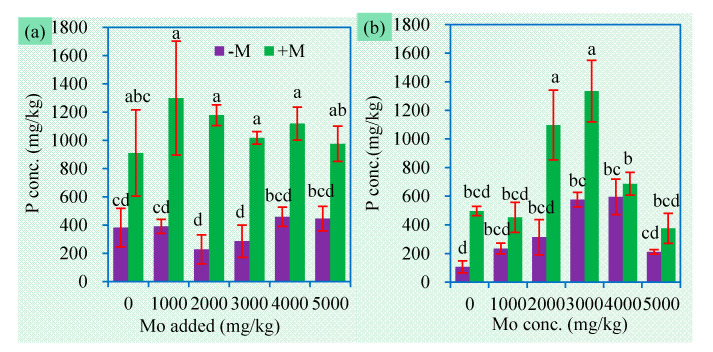
Shoot (**a**) and root (**b**) P concentrations (mean ± SE, *n* = 4) of sweet sorghum exposed to different concentrations of Mo. −M and +M represent non-AM inoculation and inoculation with *Claroideoglomus etunicatum* BEG168, respectively. Different letters over the bar represent significant differences between the means among different treatments using one-way ANOVA followed by Duncan’s multiple range test (*p* < 0.05). Two-way ANOVA results for shoot P concentrations: AM inoculation: *F* = 25.9 **, Mo: *F* = 0.3 ns, AM × Mo: *F* = 0.3 ns; two-way ANOVA results for root P concentrations: AM inoculation: *F* = 4.2 *, Mo: *F* = 0.7 ns, AM × Mo: *F* = 1.0 ns. Significance levels: * *p* < 0.05; ** *p* < 0.01; ns non-significant effect.

Mining activities not only cause increasing accumulation of mine waste and contaminants in the environment, but also produce a series of damages on soil quality, such as nutrient deficiency [13]. It is necessary to clarify the changes in plant nutrition status as influenced by MoS_2_ and AMF. As shown in Figure 5, P concentrations in shoots and root were not significantly influenced by Mo addition. Just as the most widely accepted fact, we once again confirmed AMF substantially improved plant P nutrition (Figure 5). Compared to MoO_4_^2−^, phosphate has a higher affinity for sorption sites in soils. Soil available P (H_2_PO_4_^−^ and HPO_4_^2−^) can compete with MoO_4_^2−^ for adsorption sites [44], and liberate more soil-bound Mo into the soil solution, thereby enhancing Mo uptake by plants [5]. P can also form a phosphomolybdate complex in soils, which may be taken up readily by plants [45]. It is inferred that plants might absorb molybdate through a phosphate transporter [46]. Given the excellent ability of AMF to improve plant P absorption, it is understandable that mycorrhizal plants always have higher Mo accumulation than nonmycorrhizal plants. 

**Figure 6:**
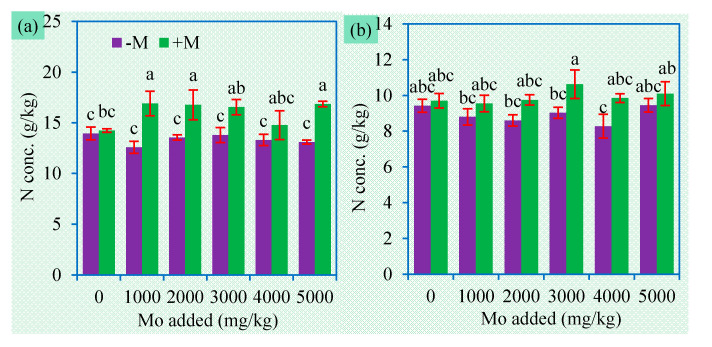
Shoot (**a**) and root (**b**) N concentrations (mean ± SE, *n* = 4) of sweet sorghum exposed to different concentrations of Mo. −M and +M represent non-AM inoculation and inoculation with *Claroideoglomus etunicatum* BEG168, respectively. Different letters over the bar represent significant differences between the means among different treatments using one-way ANOVA followed by Duncan’s multiple range test (*p* < 0.05). Two-way ANOVA results for shoot N concentrations: AM inoculation: *F* = 31.8 **, Mo: *F* = 0.9 ns, AM × Mo: *F* = 1.7 ns; two-way ANOVA results for root N concentrations: AM inoculation: *F* = 15.3 **, Mo: *F* = 1.3 ns, AM × Mo: *F* = 0.9 ns. Significance levels: * *p* < 0.05; ** *p* < 0.01; ns non-significant effect.

Mo is a component of several key enzymes including nitrogenase and nitrate reductase, and plays crucial roles in plant N metabolic processes, such as N fixation, nitrate reduction, and N transport [47]. Mo deficiency or excess can result in poor N nutrition in plants [21,48]. We found Mo addition had no significant effects on N concentrations in plant tissues (Figure 6), which can be attributed to the relatively “normal” Mo accumulation in plant tissues. Although plants did not suffer from N deficiency, AMF also improved plant N nutrition (Figure 6). Nutrient deficiency, especially macronutrients such as N, is a key factor restricting the plant establishment on mine areas. AMF may have a great potential for revegetation of mining-impacted sties with low soil fertility. 

**Figure 7:**
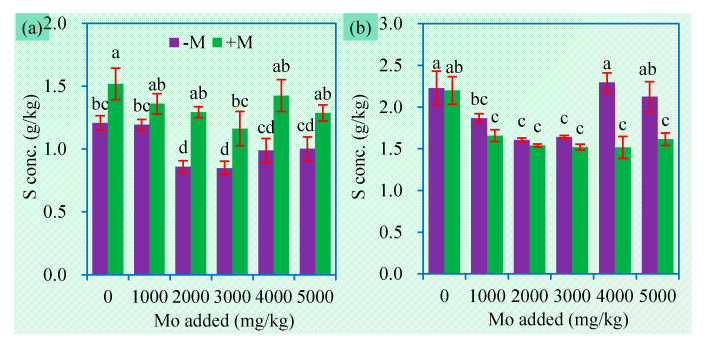
Shoot (**a**) and root (**b**) S concentrations (mean ± SE, *n* = 4) of sweet sorghum exposed to different concentrations of Mo. −M and +M represent non-AM inoculation and inoculation with *Claroideoglomus etunicatum* BEG168, respectively. Different letters over the bar represent significant differences between the means among different treatments using one-way ANOVA followed by Duncan’s multiple range test (*p* < 0.05). Two-way ANOVA results for shoot S concentrations: AM inoculation: *F* = 41.9 **, Mo: *F* = 6.7 **, AM × Mo: *F* = 1.0 ns; two-way ANOVA results for root S concentrations: AM inoculation: *F* = 18.5 **, Mo: *F* = 8.7 **, AM × Mo: *F* = 3.5 *. Significance levels: * *p* < 0.05; ** *p* < 0.01; ns non-significant effect.

Due to the presence of the S component, MoS_2_ is expected to improve plant S uptake. However, addition of MoS_2_ did not increase and even decreased S concentrations in plant tissues (Figure 7). The bioavailability of the S in MoS_2_ depends on at least two processes, i.e., the release of S^2−^ ions from MoS_2_ and their subsequent oxidation into SO_4_^2−^. MoS_2_ is difficult to dissolve and the released S^2−^ easily precipitate in soil, thereby preventing their transformation into SO_4_^2−^. Moreover, due to similar physicochemical characteristics between MoO_4_^2−^ and SO_4_^2−^, they may compete for the same transport-binding sites [16]. We can conclude that the S element in MoS_2_ is little available for plants. 

AM inoculation significantly improved shoot S concentration, but sometimes decreased root S concentrations (Figure 7). The total S uptake in plants was still enhanced by AM inoculation, due to the higher biomass of the inoculated plants (Figure 2), suggesting that AMF can improve S uptake and mediate S translocation in plants. AM fungus *Glomus intraradices* (now renamed as *Rhizophagus intraradices*) can take up reduced forms of S (cysteine and methionine) and transport them to plants [49], which implies a possibility of AMF to directly utilize the S in MoS_2_. We also found a significant interaction between AM inoculation and MoS_2_ on root Mo concentration. In addition, AMF may improve S uptake by regulating sulfate transporters in plants [50]. How AMF influence sulfide contaminants such as MoS_2_ deserves more in-depth research in the future. 

### 3.5. Chlorophyll Fluorescence Parameters

**Figure 8:**
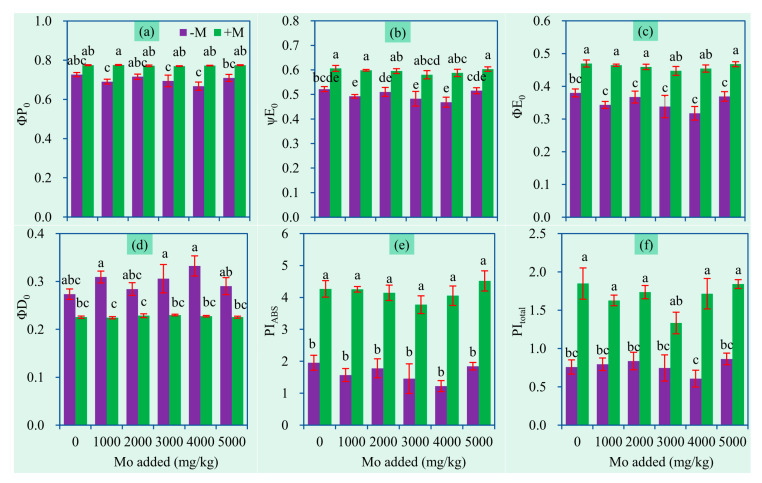
JIP-test parameters (mean ± SE, *n* = 4) of sweet sorghum exposed to different concentrations of Mo. (**a**) φPo, (**b**) ψEo, (**c**) φEo, (**d**) φDo, (**e**) PI_ABS_, (**f**) PI_total_. −M and +M represent non-AM inoculation and inoculation with *Claroideoglomus etunicatum* BEG168, respectively. Different letters over the bar represent significant differences between the means among different treatments using one-way ANOVA followed by Duncan’s multiple range test (*p* < 0.05). The definitions of the parameters were shown in Table 2 and two-way ANOVA results were shown in Table 3.

The JIP-test for fast fluorescence transients is considered an effective tool for determining mycorrhizal effects on host plants [51]. Several JIP-test parameters of chlorophyll fluorescence were significantly changed by AM inoculation (Table 3). As shown in Figure 8, AM inoculation enhanced φPo, ψEo and φEo, but decreased φDo, which suggests that the leaves of the inoculated plants have higher maximum quantum efficiency and quantum yield for electron transport, and lower quantum yield for energy dissipation. Particularly, mycorrhizal plants had much higher performance index (PI) than nonmycorrhizal ones. These changes imply that AMF can decrease energy dissipation and increase comprehensive photosynthesis performance, and consequently enhance the efficiency of PSII photochemical activities of inoculated plants. Rai et al. [51] found that maize plants inoculated with *Piriformospora indica* and mixed AMF exhibited relatively higher quantum yield compared to nonmycorrhizal plants, and electron flow yield (φEo = φPo × ψEo) was highly responsive to AM inoculation. Our results are in agreement with numerous findings that mycorrhizal plants generally can achieve a higher photosynthesis rate via modulating chlorophyll fluorescence parameters, and thereby can grow better under environmental stress such as high temperature, salt stress, and metal toxicity [52,53,54,55]. An essential nutrient like P is indispensable for photosynthesis processes such as photosynthetic phosphorylation. Nutrient deficiency can affect phytochemical processes [26]. AM plants generally have higher photosynthetic capacity and leaf nutrient concentrations [56]. Putatively, AMF-improved P nutrition, as well as N and S, can partially explain the higher photochemical activities. 

In addition, Mo did not influence the target JIP-test parameters (Figure 8), indicating that Mo did not produce damages in photosynthetic properties. Excess toxic metals often inhibit plant photochemical activity and plant growth [54,57]. On the contrary, our results indicate that Mo is of low phytotoxicity. This is in accordance with the observations on unchanged biomass production in the plants exposed to Mo (Figure 2). Plants were shown to sequestrate excessive Mo in vacuoles, thereby reducing Mo toxicity and damages [58].

## 4. Conclusions

Unlike soluble molybdate, MoS_2_ at the test concentrations (1000–5000 mg/kg) produced no obvious phytotoxic effects on sweet sorghum growth and AM colonization, but resulted in high Mo accumulation in plant tissues, implying potential health risks for humans and animals. AM inoculation always increased Mo concentrations and uptake of the plants exposed to Mo with different doses, but did not increase Mo phytotoxicity, suggesting mycorrhizal plants may have a preference for Mo and higher tolerance. AMF improved the performance of sweet sorghum and the biomass production in Mo-contaminated soil via enhancing nutrient uptakes of P, N and S, and photosynthesis efficiency. In conclusion, MoS_2_ has low phytotoxicity to sweet sorghum and AMF, and sweet sorghum together with AMF can be used for phytoremediation and revegetation of Mo-contaminated farmland and Mo-mine disturbed areas, as well as biomass production for biofuel on such sites. 

## Figures and Tables

**Table 1:** Physicochemical properties of the soil used in the present study.

pH	Organic Matter	Total N	Total P	Total K	Total Mo	Total S
7.32	7.6%	13 g/kg	337.3 mg/kg	2.7 g/kg	8.4 mg/kg	147.6 mg/kg

**Table 2:** Definitions of terms for calculation of the JIP-test parameters from the chlorophyll, a fluorescence transient OJIP emitted by dark-adapted leaves.

Parameter	Definition
φPo	The maximum quantum efficiency of PSII
ψEo	Efficiency/probability that an electron moves further than reduced Q_A_ (primary electron acceptor of PSII)
φEo	Quantum yield of electron transport
φDo	Quantum yield of energy dissipation
PI_ABS_	Performance index (potential) for energy conservation from exciton to reduction of intersystem electron acceptors
PI_tota_l	Performance index (potential) for energy conservation from exciton to reduction of PSI end acceptors

**Table 3:** Two-way ANOVA results from AM inoculation, Mo addition concentration and their interactions the JIP-test parameters.

Parameter	AM Inoculation	Mo	AM × Mo
φPo	89.14 **	1.31 ns	1.22 ns
ψEo	121.40 **	1.86 ns	0.4 ns
φEo	142.09 **	1.86 ns	0.68 ns
φDo	89.14 **	1.31 ns	1.22 ns
PI_ABS_	272.70 **	1.58 ns	0.36 ns
PI_total_	157.18 **	1.62 ns	1.16 ns

Significance levels: ** *p* < 0.01; ns non-significant effect.

## Supplementary Materials

Supplementary materials can be found at https://www.mdpi.com/2309-608X/6/2/44/s1. Table S1. Pearson correlation between Mo concentrations in soil and Mo concentrations/uptake in plant tissues.
