# Supplementary material for: Arbuscular Mycorrhizal Fungi Improve the Performance of Sweet Sorghum Grown in a Mo-Contaminated Soil
Source: J Fungi (Basel). 2020 Mar 31;6(2):44. doi: 10.3390/jof6020044 (PMC7344874; doi:10.3390/jof6020044)
Supplement: Supplementary file 1 [file jof-06-00044-s001.pdf]

Table S1. Pearson correlation between Mo concentrations in soil and Mo concentrations/uptake in plant tissues.

	**Shoot Mo conc.**		**Root Mo conc.**		**Shoot Mo uptake**		**Shoot Mo uptake**	
	**-M**	**+M**	**-M**	**+M**	**-M**	**+M**	**-M**	**+M**
Soil Mo conc.	0.584ns	0.835*	0.955**	0.847*	0.419ns	0.838*	0.930**	0.878*

Significance levels: *$P < 0.05$; **$P < 0.01$; ns non-significant effect.
